# The Principles and Practice of Obstetric Medicine and Surgery, in Reference to the Process of Parturition

**Published:** 1855-04

**Authors:** 


ART. IX.—*The Principles and Practice of Obstetric Medicine and Surgery, in reference to the Process of Parturition. With* 64 *Plates and numerous Wood Cuts.* By FRANCIS H. RAMSBOTHAM, M. D., etc., etc. A new American Edition, revised by the Author. With Notes and Additions, by WILLIAM V. KEATING, A. M., M. D. Lecturer, etc. Philadelphia: Blanchard and Lea. 1855.

A friend, much more competent than ourselves, has promised us an analysis of this work, and we need therefore only to announce a new edition of a standard work, with the remark that it is typographically excellent, and does great credit to the publishers. H.

